# Enhancing Interstitial Lung Disease Diagnoses Through Multimodal AI Integration of Histopathological and CT Image Data

**DOI:** 10.1111/resp.70036

**Published:** 2025-04-02

**Authors:** Kris Lami, Mutsumi Ozasa, Xiangqian Che, Wataru Uegami, Yoshihiro Kato, Yoshiaki Zaizen, Naoko Tsuyama, Ichiro Mori, Shin Ichihara, Han‐Seung Yoon, Ryoko Egashira, Kensuke Kataoka, Takeshi Johkoh, Yasuhiro Kondo, Richard Attanoos, Alberto Cavazza, Alberto M. Marchevsky, Frank Schneider, Jaroslaw Wojciech Augustyniak, Amna Almutrafi, Alexandre Todorovic Fabro, Luka Brcic, Anja C. Roden, Maxwell Smith, Andre Moreira, Junya Fukuoka

**Affiliations:** ^1^ Department of Pathology Informatics Nagasaki University Graduate School of Biomedical Sciences Nagasaki Japan; ^2^ Department of Respiratory Medicine Nagasaki University Graduate School of Biomedical Sciences Nagasaki Japan; ^3^ Strategic AI Group Future Corporation Tokyo Japan; ^4^ Department of Pathology Kameda Medical Center Kamogawa Japan; ^5^ Division of Respirology, Neurology and Rheumatology, Department of Medicine Kurume University School of Medicine Kurume Japan; ^6^ Division of Pathology Cancer Institute, Japanese Foundation for Cancer Research Tokyo Japan; ^7^ Department of Pathology International University of Health and Welfare Narita Japan; ^8^ Department of Surgical Pathology Sapporo Kosei General Hospital Sapporo Japan; ^9^ Department of Radiology Saga University Saga Japan; ^10^ Department of Respiratory Medicine and Allergy Tosei General Hospital Seto Japan; ^11^ Department of Radiology Kansai Rosai Hospital Amagasaki Japan; ^12^ Department of Thoracic Surgery Kitasato University School of Medicine Kanagawa Japan; ^13^ Department of Cellular Pathology Cardiff University Cardiff UK; ^14^ Pathology Unit Azienda USL/IRCCS di Reggio Emilia Reggio Emilia Italy; ^15^ Department of Pathology Cedars‐Sinai Medical Center Los Angeles California USA; ^16^ Department of Pathology and Laboratory Medicine Emory University Atlanta Georgia USA; ^17^ Institut for Pathology Spandau Berlin Germany; ^18^ Department of Anatomical Pathology King Fahad Medical City Riyadh Saudi Arabia; ^19^ Department of Pathology and Legal Medicine Ribeirão Preto Medical School University of São Paulo São Paulo Brazil; ^20^ Diagnostic and Research Institute of Pathology Medical University of Graz Graz Austria; ^21^ Department of Laboratory Medicine and Pathology Mayo Clinic Rochester Minnesota USA; ^22^ Department of Laboratory Medicine and Pathology Mayo Clinic Scottsdale Arizona USA; ^23^ Department of Pathology NYU Grossman School of Medicine New York New York USA

**Keywords:** CT, histopathology, interstitial lung disease, multimodal AI, UIP

## Abstract

**Background and Objective:**

The diagnosis of interstitial lung diseases (ILDs) often relies on the integration of various clinical, radiological, and histopathological data. Achieving high diagnostic accuracy in ILDs, particularly for distinguishing usual interstitial pneumonia (UIP), is challenging and requires a multidisciplinary approach. Therefore, this study aimed to develop a multimodal artificial intelligence (AI) algorithm that combines computed tomography (CT) and histopathological images to improve the accuracy and consistency of UIP diagnosis.

**Methods:**

A dataset of CT and pathological images from 324 patients with ILD between 2009 and 2021 was collected. The CT component of the model was trained to identify 28 different radiological features. The pathological counterpart was developed in our previous study. A total of 114 samples were selected and used for testing the multimodal AI model. The performance of the multimodal AI was assessed through comparisons with expert pathologists and general pathologists.

**Results:**

The developed multimodal AI demonstrated a substantial improvement in distinguishing UIP from non‐UIP, achieving an AUC of 0.92. When applied by general pathologists, the diagnostic agreement rate improved significantly, with a post‐model κ score of 0.737 compared to 0.273 pre‐model integration. Additionally, the diagnostic consensus rate with expert pulmonary pathologists increased from κ scores of 0.278–0.53 to 0.474–0.602 post‐model integration. The model also increased diagnostic confidence among general pathologists.

**Conclusion:**

Combining CT and histopathological images, the multimodal AI algorithm enhances pathologists' diagnostic accuracy, consistency, and confidence in identifying UIP, even in cases where specialised expertise is limited.

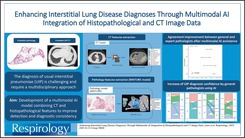

## Graphical abstract

**See related** editorial

**Summary**

- We aimed to develop a multimodal artificial intelligence (AI) model combining CT and pathological images for a more accurate diagnosis of interstitial lung diseases (ILDs), particularly usual interstitial pneumonia (UIP).
- The AI achieved an AUC of 0.92 in distinguishing UIP from non‐UIP cases and significantly improved pathologists' diagnostic confidence.

## 1 Introduction

Interstitial lung disease (ILD) comprises a heterogeneous group of disorders primarily characterised by fibrosis of the lung interstitium and inflammation [1]. The incidence of these disorders varies widely, owing to the rarity of certain entities and the high frequency of others [2], making encounters with ILD cases not uncommon for general pathologists in daily clinical practice. However, achieving a precise diagnosis of ILD and identifying the causative agent/condition demands a high level of expertise due to the overlapping histological features, radiological presentations, and clinical symptoms [3]. Notably, many challenging ILD cases that cannot be diagnosed based solely on clinical and radiographic findings necessitate surgical lung biopsy and histopathological evaluation, further complicating the diagnostic process. For most ILDs, a conclusive diagnosis demands integrating clinical, radiological, and pathological features, which calls for a multi‐disciplinary discussion (MDD) [4, 5]. As a result, diagnosing ILD remains extremely challenging and is often deemed esoteric for most non‐pulmonary pathologists.

The recognition of the usual interstitial pneumonia (UIP) histological pattern, which is present in numerous types of ILD, plays a crucial role as an indicator of potential fibrosis progression in the future and is associated with poor prognosis [5, 6]. Nevertheless, it is widely acknowledged that diagnosing interstitial pneumonia, including UIP, remains inconsistent even among medical specialists proficient in both histopathological and radiographic image evaluation [7, 8, 9]. This inconsistency poses significant challenges for pathologists who do not routinely encounter interstitial pneumonia cases, making it extremely difficult for them to conduct accurate tissue evaluation, including computed tomography (CT) image analysis/interpretation. Consequently, many cases of interstitial pneumonia tend to be concentrated in facilities with specialised departments equipped to handle such complex diagnoses.

To address this situation and improve the accessibility of ILD diagnoses, we have previously developed MIXTURE, an explainable artificial intelligence (AI) strategy that relies on extracting histopathological features evaluated by specialist pathologists through the use of a deep learning algorithm [10]. This approach has been applied explicitly to ILDs, focusing on predicting the diagnosis of UIP. Notably, the model demonstrated outstanding performance with an area under the curve (AUC) of 0.86 and showed promising results in predicting other ILDs as well.

Despite the high performance of the MIXTURE model, an AI model relying solely on histopathological information may not provide sufficient depth for achieving an objective diagnosis. ILDs pose a diagnostic challenge requiring a multi‐disciplinary approach to reach a definitive diagnosis. A potential solution lies in integrating radiological or clinical data into AI models to mimic real‐life conditions. The growing trend of employing multimodal AI models, which combine radiological and pathological images to attain final diagnoses, is evident across various medical disciplines. Notably, similar approaches have demonstrated successful outcomes in classifying gliomas [11, 12] and lung cancer [13].

To enhance the realism and objectivity of ILD diagnoses, we developed a multimodal AI model that combines histopathological and CT image data. This integrated approach aims to achieve a more effective prediction of UIP, irrespective of the pathologist's expertise level. By fusing both histopathological and radiological information, our AI model holds the potential to provide more accurate and reliable predictions, enhancing the diagnostic process for ILD, particularly in cases of UIP.

## 2 Methods

### 2.1 Study Design

**FIGURE 1:**
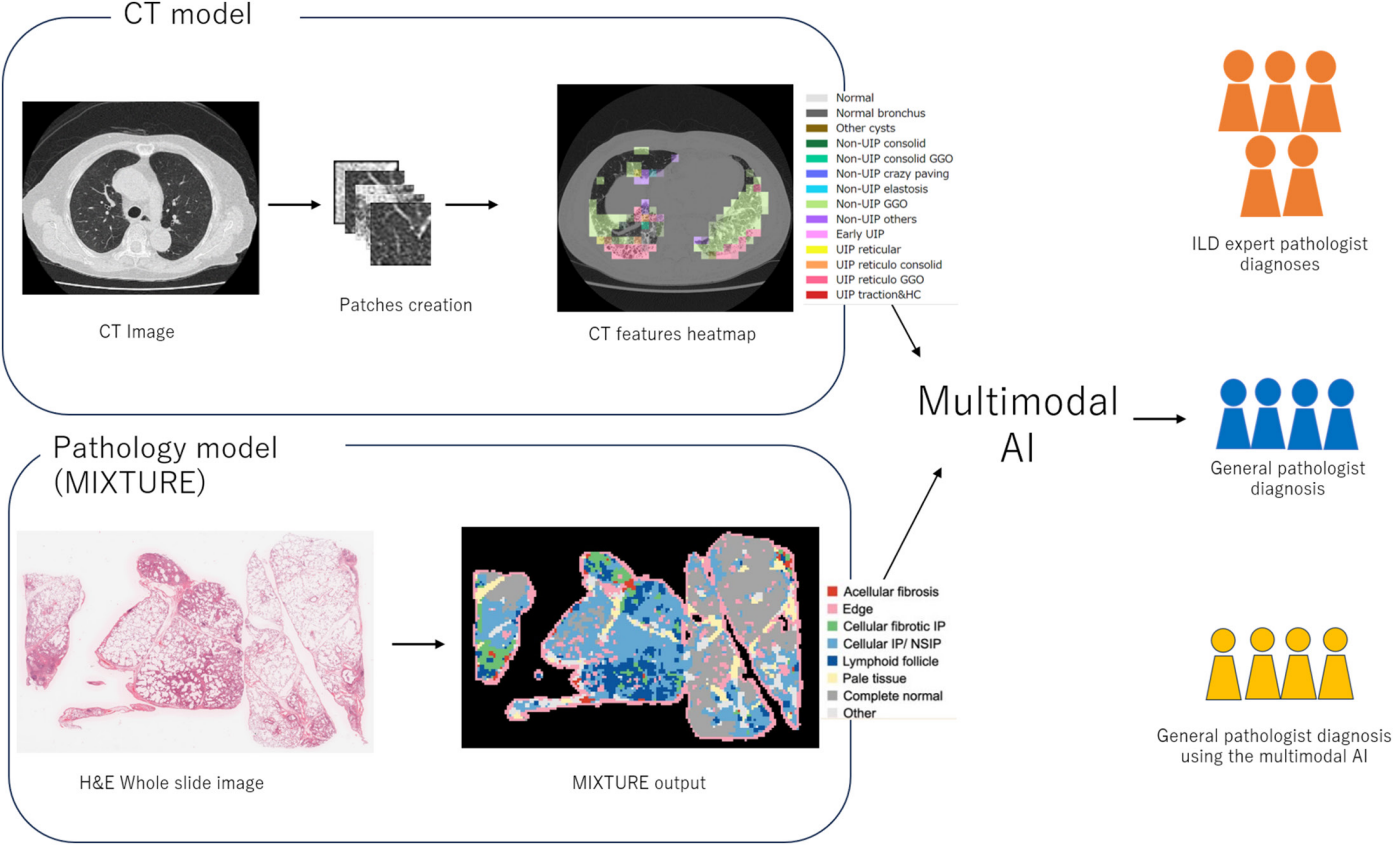
Workflow of the study. The multimodal AI algorithm was developed by integrating computed tomography and histopathological components, both designed to detect the presence of usual interstitial pneumonia. Subsequently, the model's performance was evaluated by comparing it to that of a pulmonary pathologist with expertise in interstitial lung disease, as well as to general pathologists before and after utilising the model.

The study received approval from the Institutional Review Board of Nagasaki University Hospital (Approval number 22092203) to develop a multimodal AI system for accurately diagnosing UIP using a retrospective case collection approach. The multimodal AI algorithm was designed by combining models trained on CT and pathological images, enabling the prediction of UIP presence. The performance of this AI model was then compared to that of pulmonary pathologists and general pathologists, with and without the aid of the AI model, to evaluate its impact on diagnostic accuracy. Figure 1 illustrates the schematic workflow of the study.

### 2.2 Data Collection

**FIGURE 2:**
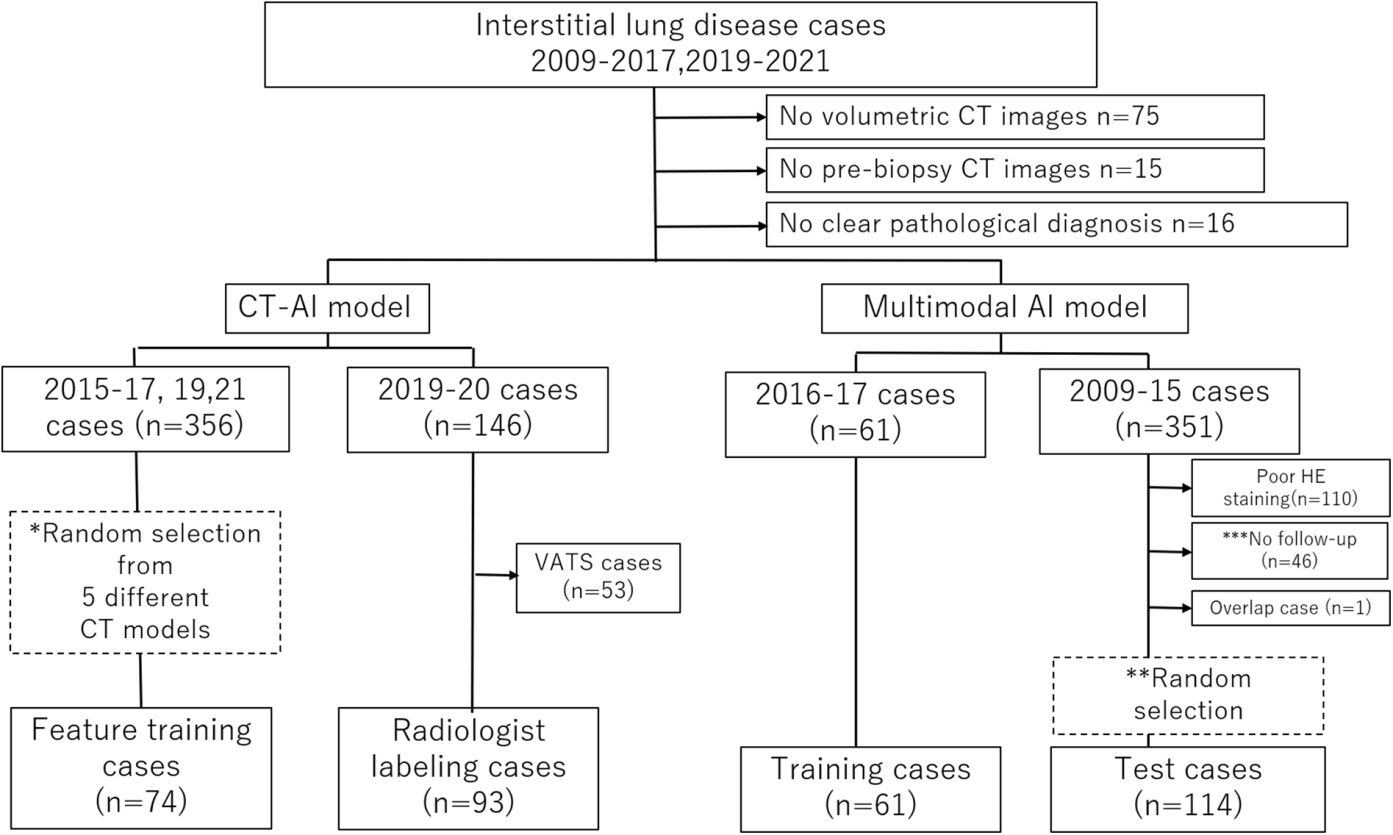
Cases distribution. A total of 74 interstitial lung disease cases were used for feature extraction from computed tomography images, while 93 cases were employed for annotation. The multimodal AI model was trained using 61 cases and subsequently tested on 114 cases.

A cohort comprising 770 cases diagnosed with ILD after MDD was chosen from a single institution (Tosei General Hospital, Seto, Japan) covering the period from 2009 to 2021. To ensure a robust dataset, cases with no volumetric CT images, pre‐biopsy CT images, and no precise pathological diagnosis were excluded (*n* = 106). All remaining cases included CT images without a definitive UIP pattern and with corresponding pathological tissue samples. After a random selection to narrow down the cohort, the remaining cases (*n* = 324) were partitioned as follows: 74 cases for extracting CT image features, 93 cases for creating the AI model using CT image features; additionally, 61 cases were used for multimodal AI model training, and 114 cases for its testing. A detailed representation of the case distribution is illustrated in Figure 2. All histopathological slides were scanned using the digital slide scanner Aperio ScanScope CS2 (Leica Biosystems, Buffalo Grove, IL) to produce whole slide images (WSIs).

### 2.3 Creation of Pathology AI That Recognises UIP With the MIXTURE Strategy

Regarding the histopathological component of the multimodal AI model, hereinafter referred to as Pathology‐AI, the intermediate output of the MIXTURE model was used [10]. The model is able to predict the presence of UIP in video‐assisted thoracic surgery (VATS) histopathological specimens. A comprehensive account of the creation of Pathology‐AI, along with an in‐depth analysis of its performance, can be found in our prior publication [10].

### 2.4 Creation of CT‐AI That Recognises UIP

#### 2.4.1 Features Extraction

**FIGURE 3:**
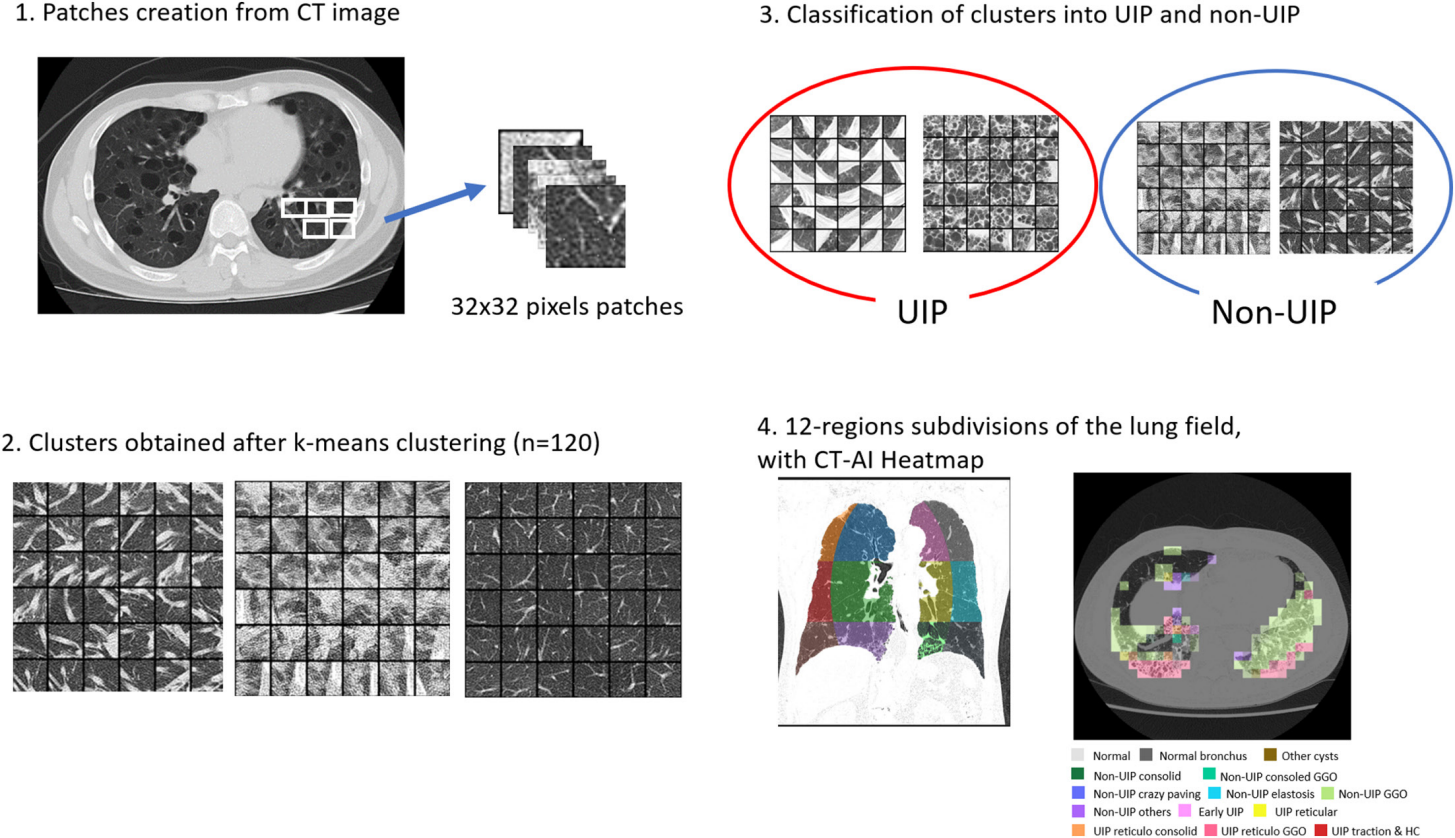
Development of the computed tomography counterpart of the multimodal AI model, CT‐AI. 1. Image segmentation of the CT image's lung field produced over a million patches of 32 × 32 pixels. 2. Some examples of the 120 clusters obtained from k‐means clustering, emphasising feature similarity between different patches 3. Clusters were further classified into 8 distinct radiological patterns by 2 pathologists to be finally regrouped under UIP or non‐UIP 4. 12‐regions division of the lung on the left coupled with an example of results of the CT‐AI on the right.

The radiological counterpart of the multimodal AI, referred to as CT‐AI, was developed using CT images from 74 patients diagnosed with chronic interstitial pneumonia, acquired with thin slices (0.5–0.6 mm) from multiple CT scanner models. Lung segmentation was performed using the lungmask library [14], generating 574,977 patches divided into 32 × 32 pixels with a 48 × 48 stride (Figure 3(1)).

Features extraction was conducted using an automated, self‐supervised learning approach with the SimSiam library, transforming patches into 2048‐dimensional feature vectors (Figure 3) [15]. No adjustments for class imbalances were required. From these, 74,970 feature vectors were clustered into 120 groups using spherical k‐means. Two ILD specialists (M.O. and Y.Z.) classified these clusters into 8 patterns, including UIP and non‐UIP categories, based on image features and spatial localisation (Figure 3(3)).

The lung field was further divided into 12 anatomical regions (left–right, upper‐middle‐lower, inner‐outer), and 28 features were extracted: 24 based on the proportions of the 8 patterns in the upper, middle, and lower regions, 1 capturing the difference in normal lung proportions between left and right, and 3 representing spatial variations in UIP patterns between inner and outer regions (Figure 3(4)). Further details are provided in Supporting Information.

#### 2.4.2 UIP Labelling of CT Images

To train and test the CT‐AI model, 93 consecutive CT images from patients with chronic interstitial pneumonia who had not undergone VATS were retrospectively collected from 2019 to 2020. Two ILD‐expert radiologists (R.E. and T.J.) assessed the likelihood of UIP presence in 10% increments (0%–100%). The average UIP probability was calculated for each case, and cases with 50% abnormalities were assigned a pseudo‐consensus UIP label. Based on these evaluations, each case was classified as either UIP or non‐UIP.

#### 2.4.3 Creation of CT‐AI

The 93 annotated CT image cases were randomly split into two sets: a training set of 46 and a testing set of 47 cases. From the training set, a random forest model was developed (scikit‐learn, version 0.24.2) capable of predicting the presence of a UIP based on the 28 features that were previously created. The model generated a continuous variable output ranging from 0 to 1, with values closer to 1 indicating a higher likelihood of UIP. To dichotomise the predictions into UIP and non‐UIP categories, the cutoff point was set at 0.5, where values equal to or above 0.5 were classified as UIP, while those below 0.5 were labelled as non‐UIP. Further details on the creation of the CT‐AI are provided in the supplemental material.

### 2.5 Creation of the Multimodal AI Model Combining Histopathological and CT Images

#### 2.5.1 Training

A predictive model was developed to determine the histological diagnosis of UIP using pathological features from the MIXTURE model [10], 0–1 continuous variables from CT‐AI, and CT image features. The multimodal AI produced continuous values (0–1), where values closer to 1 indicated a higher likelihood of UIP. A cohort of 70 VATS patients (2016–2017) was enrolled, and after excluding 9 cases without chest CT images or with unclassifiable histopathology, 61 cases were used for model training via a Random Forest strategy. Parameters of the trained model are available in the supplemental material.

#### 2.5.2 Testing

Between 2009 and 2015, 351 patients who underwent VATS were identified for the test set. After excluding 110 cases with poor HE staining and 15 unclassifiable cases, 226 cases were evaluated by a specialised ILD pathologist (J.F.), classifying 136 as UIP and 90 as non‐UIP. An additional 46 cases with insufficient follow‐up data were also excluded. From the remaining cases, 120 (60 UIP, 60 non‐UIP) were selected, ensuring a balanced distribution. After further exclusions, 114 cases were finalised for model testing.

#### 2.5.3 Validation by General Pathologists

From the test cohort of 114 cases, 69 were randomly selected to assess the performance of the multimodal AI with general pathologists. Four pathologists (N.T., I.M., S.I., and H.S.Y.), without ILD expertise, reviewed the haematoxylin and eosin (H&E) and chest CT images, diagnosing UIP/non‐UIP and indicating confidence levels (high, medium, low). After initial diagnoses, they were presented with the multimodal AI results and asked to reassess the cases.

Additionally, 11 ILD specialists (R.A., A.C., A.M.M., F.S., J.W.A., A.A., A.T.F., L.B., A.C.R., M.S., and A.M.) evaluated the same 69 cases to establish a “consensus diagnosis,” which served as the ground truth. The agreement rate between the general pathologists' diagnoses and the expert consensus was calculated before and after AI assistance, along with changes in diagnostic confidence.

### 2.6 Statistical Analysis

Cohen's Kappa was used to calculate diagnostic concordance rates among the four raters both before and after the input of the multimodal AI, as well as to assess their concordance with the diagnoses made by expert pulmonary pathologists. Area under the curve (AUC), precision, recall, and F1 score were used to evaluate the performance of the AI. This study used R (v. 4.3.0.) as the statistical software.

## 3 Results

### 3.1 Clinicopathological Characteristics of Patients

This study included cases diagnosed with interstitial lung disease who underwent VATS between 2009 and 2021. Of these cases, those without an accurate pathology diagnosis, those without 0.5 mm slice chest CT images, and those without pathology specimens collected simultaneously as CT imaging were excluded. Finally, 74 and 93 cases were used for feature extraction and the creation of the CT‐AI, respectively; 61 cases were used for training to create the multimodal AI, and 114 cases were used for testing (Figure 2). Age, sex ratio, and the number of UIP cases in the training and testing cohorts can be found in Table 1.

**TABLE 1:** Clinicopathological characteristics of the cohort.

	Learning of CT image features, *n* = 74	Training of the multimodal AI, *n* = 61	Testing of the multimodal AI, *n* = 114
Age, mean (intervals)	65 (33–81)	61 (30–75)	63 (31–75)
Male (%)	48 (65%)	38 (62%)	62 (54%)
Pathology label UIP:non UIP	n/a	34:27	56:58

### 3.2 UIP Prediction Results With CT‐AI

The development of the multimodal AI algorithm for UIP classification involved the creation of distinct AI components, notably the CT‐AI and the Pathology‐AI. The performance outcomes of the Pathology‐AI, facilitated by the MIXTURE model, are comprehensively detailed in our previous publication [10]. Furthermore, the CT‐AI, based on the random forest model, demonstrated an accuracy of 78.51% ± 4.09% in the dichotomised classification of UIP and non‐UIP within lung CT images. This classification was accompanied by recall, specificity, precision, and F1 scores, which stood at 89.60% ± 5.21%, 60.04% ± 12.88%, 79.93% ± 6.27%, and 84.18% ± 2.92%, respectively. When juxtaposed against the labels provided by radiologists, the CT‐AI model exhibited a high AUC of 0.8492 ± 0.0423 for the UIP classification.

### 3.3 UIP Prediction Results With Multimodal AI

Next, the assessment of the multimodal AI, integrating both the CT images component and the histopathological component, was conducted within a cohort comprising 114 patients. In this evaluation, the model demonstrated its proficiency by accurately detecting the presence of UIP within the test set, achieving an overall accuracy rate of 82.37% ± 2.81%. This accomplishment was further substantiated by recall and specificity values of 93.08% ± 2.14% and 73.39% ± 5.11%, respectively, precision of 74.76% ± 3.68%, and an F1 score of 82.85% ± 2.36%. For example, within the test set, three cases were diagnosed with chronic hypersensitivity pneumonitis (CHP), including one case with an uncertain diagnosis (CHP or idiopathic pulmonary fibrosis) from the MDD. The AI classified two of the CHP cases as non‐UIP, while the uncertain case was recognized as UIP.

To ascertain the model's performance against a robust reference, the UIP/non‐UIP binary classifications rendered by the pulmonary pathologist (J.F.) were considered the ground truth. In this context, the multimodal AI exhibited commendable discriminative power, as evidenced by an AUC of 0.9238 ± 0.0152 for detecting UIP, with a confidence interval of 0.9123 to 0.9353.

### 3.4 Classification Performance of General Pathologists Using the Multimodal AI

**FIGURE 4:**
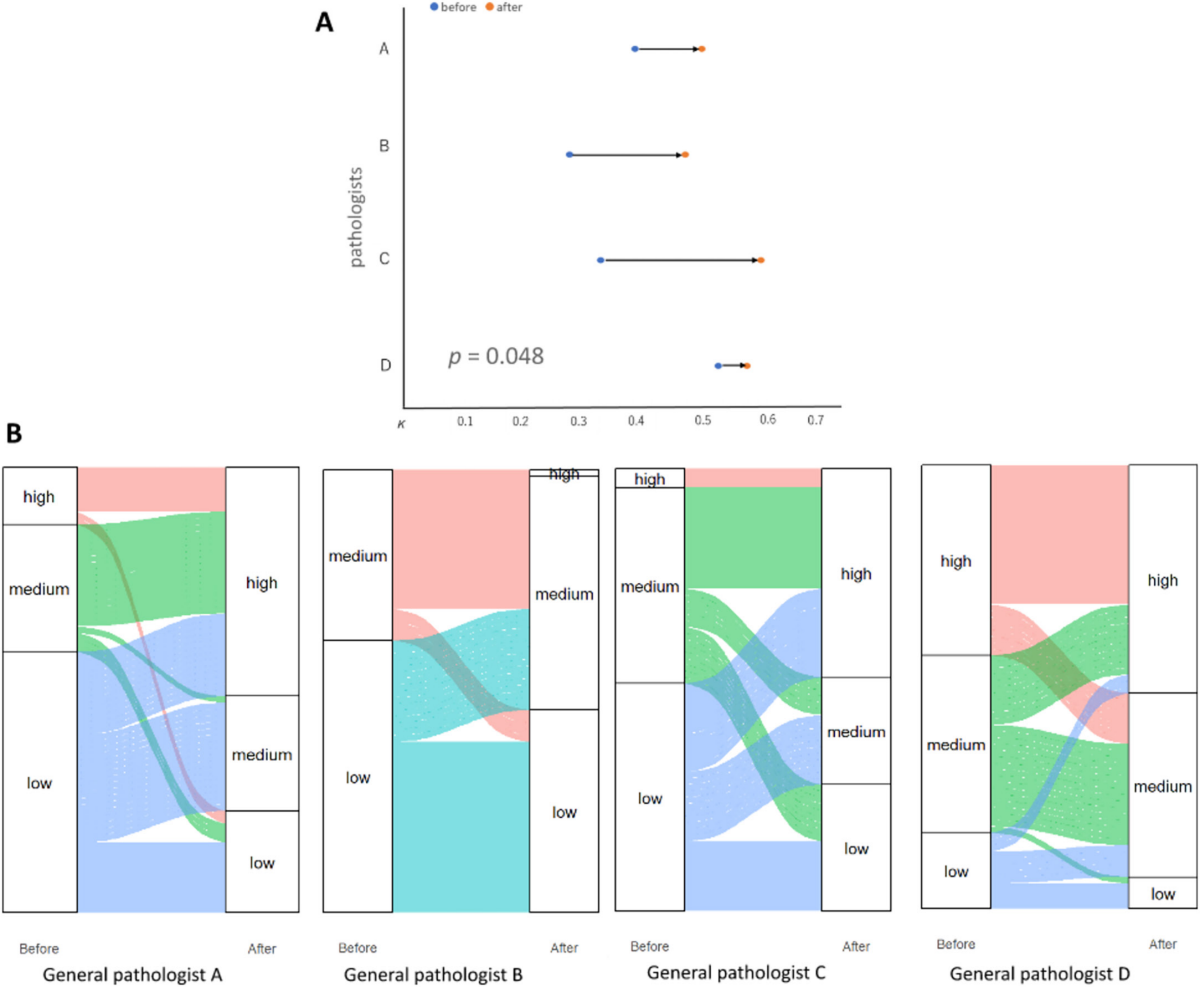
Impact of the multimodal AI on general pathologists. (A) Diagnostic concordance between general pathologists and consensus diagnosis, before and after exposure to the multimodal AI results. Overall improvement in concordance was noted after pathologists were aware of the results of the multimodal AI model. (B) Diagnostic confidence assessment for general pathologists before and after AI results. Improvement in diagnostic confidence was observed in 3 out of 4 pathologists, with 50% of cases diagnosed with high confidence after consulting the multimodal AI results.

The impact of the multimodal AI algorithm on the diagnostic accuracy of general pathologists was assessed using 69 VATS specimens. Ground‐truth diagnoses were determined by consensus among 11 pulmonary pathologists. Four general pathologists, without ILD expertise, classified cases as UIP/non‐UIP and rated their confidence levels. Before using the AI, the diagnostic concordance among general pathologists was low, with a κ coefficient of 0.273, and agreement with the expert consensus was fair to moderate (κ = 0.278 to 0.53, Figure 4A). Diagnostic confidence was also limited, with high confidence in only about 10% of cases (Figure 4B). After using the AI, diagnostic concordance improved significantly, with a κ value of 0.737 among general pathologists and 0.474 to 0.602 in agreement with the expert consensus, with a p‐value of 0.048, indicating a statistical significance (Figure 4A). Diagnostic confidence also increased, with three of the four general pathologists expressing high confidence in about 50% of cases (Figure 4B).

## 4 Discussion

The present study detailed the creation of a multimodal AI algorithm that integrates both CT images and histopathological images to discern UIP effectively. The radiological component of the model underwent training to identify 28 distinct features associated with the presence or absence of UIP, while the pathological AI component was developed in a prior investigation [10]. When assessed on an independent dataset, the multimodal AI algorithms demonstrated a remarkable AUC for UIP detection. Furthermore, the model's training significantly enhanced diagnostic congruence with expert pulmonary pathologists and increased the diagnostic confidence of general pathologists when evaluating UIP diagnoses. To the best of our knowledge, this study presents the first attempt to combine CT and pathological images to build an AI algorithm capable of distinguishing UIP. Moreover, the development and implementation of these multimodal AI algorithms represent a promising advancement in the accurate and confident diagnosis of UIP. By integrating CT and pathological features into an interpretable prediction framework, these algorithms hold the promise of reducing interobserver variability while likely enhancing diagnostic consistency and confidence. While not intended to replace MDD, this tool has the potential to significantly impact ILD assessment and management by supporting pathologists in their decision‐making process.

In recent years, significant advancements in AI‐based image diagnosis have been made, particularly in the realms of radiological images and histopathology [16, 17, 18, 19, 20, 21]. However, it is worth noting that many of these AI developments have been concentrated in fields characterised by well‐defined diagnostic criteria, such as tumours and infectious diseases [22, 23]. In contrast, for conditions like interstitial pneumonia, where diagnosis often relies on the subjective judgement of the evaluator and where the availability of comprehensive and accurate data for AI is limited, constructing effective AI models poses unique challenges. In response to these challenges, our previous work led to the development of the MIXTURE model, which can recognise histological UIP [10]. In our ongoing efforts to enhance the accuracy of the MIXTURE model, we have now introduced a novel approach: a multimodal AI that incorporates radiographic image characteristics to aid in diagnosing histological UIP.

Integrating clinical observations, radiographic data, and occasionally genomic characteristics with histopathological images enhances diagnostic accuracy [24, 25]. Recent advancements in machine learning have driven the development of multimodal AI systems that combine diverse diagnostic inputs to improve precision. For example, combining pathological and radiographic data has shown improved performance in glioma diagnosis [11, 12] and more accurate prognostication in high‐grade serous carcinoma of the ovary [26]. In ILDs, a multidisciplinary approach is essential, with radiographic images playing a key role in providing a comprehensive view of lesions [27, 28, 29]. Integrating a chest CT AI model into the MIXTURE model, which focuses on histopathological morphology, enables a holistic approach that improves the accuracy of histological UIP diagnosis and holds promise for other complex medical conditions.

The resulting model exhibited a notable increase in AUC compared to the performance of the MIXTURE model [10]. Specifically, the Pathology‐AI demonstrated an AUC of 0.88 for UIP prediction on a test set, using findings extracted at 2×, 5×, and 20× magnification. In contrast, the multimodal AI model in this study achieved an AUC of 0.92 for the same task, signifying a substantial enhancement in accuracy. This result is also superior to a similar developed model but only focusing on the UIP prediction on CT images, with an AUC of 0.87 [30]. Furthermore, when the multimodal AI model was employed by four pathologists who do not routinely diagnose ILDs, their diagnostic agreement rate saw significant improvements. Prior to AI integration, the κ score stood at 0.273, but following the model's inclusion, this score surged to 0.737. Moreover, the diagnostic consensus rate with expert pulmonary pathologists, initially low with κ scores ranging from 0.278 to 0.53 before AI utilisation, experienced a marked enhancement, elevating κ scores to the range of 0.474 to 0.602 post‐model integration. Additionally, using the model increased diagnostic confidence among the pathologists, with three general pathologists now exhibiting a 50% high‐confidence diagnostic rate compared to the previous 10%. These compelling results suggest that the model developed in this study holds the potential to standardise diagnoses across diverse patient populations, even in scenarios where medical professionals lack specialised expertise. Although the model was initially designed for VATS specimens, there is potential to extend this methodology to cryobiopsy, thereby creating a more versatile diagnostic tool. While our study integrates CT data into pathological diagnosis, it does not suggest modifying current radiological practices. Biopsy is not recommended for cases with a definitive UIP pattern on CT, as the multimodal AI primarily aids in inconclusive cases.

Conversely, despite its immense value, AI diagnosis is not infallible, necessitating the development of mechanisms that allow users to assess the reliability of AI‐generated judgments. Consequently, explainability holds profound significance within the medical AI field. This study's model was crafted to serve as an assisting tool for pathologists. It was designed to convey the distribution of pathological findings in a manner that enables pathologists to make informed decisions while concurrently verifying the AI's accuracy in recognising lesions. Additionally, acknowledging that pathologists may have little expertise in interpreting radiographic images, the model represents the presence of UIP as a numerical value, thereby enhancing interpretability. Pathologists can seamlessly collaborate with the AI through these thoughtful design choices, conducting diagnoses while naturally confirming the AI's performance. The integration of the AI into the clinical workflow is efficient, as the AI‐generated results can be incorporated into an existing diagnostic platform, assisting pathologists in generating more standardised reports prior to their inclusion in multidisciplinary discussions.

Several limitations have to be considered in this study. Firstly, the multimodal AI model training and evaluation were limited to cases from a single medical institution, which may affect the model's generalizability. To address this limitation, data augmentation was applied to simulate different laboratory settings and staining differences that might arise from cases originating from different institution. However, the performance of the multimodal AI should be interpreted with caution, as no external test set was used to validate its performance on an institution‐independent dataset. This limitation is primarily due to the nature of the study, which serves as a proof‐of‐concept for the multimodal AI. This issue should be addressed in a follow‐up study involving cases from multiple institutions, with ground truth established through a consensus of multiple pathologists, to comprehensively evaluate the model's performance. Secondly, although the model underwent training involving multiple CT scanner models, the histopathological tissue analysis was restricted to using a single digital slide scanner and included only VATS specimens. Thirdly, in a highly complex field such as ILD and UIP, the annotations provided by pathologists and radiologists to establish the ground truth may have introduced some degree of bias, potentially affecting the reliability of the reference standard. To mitigate this concern, a consensus‐based approach was adopted, where the ground truth diagnosis was established only when agreement was reached among a panel of expert pathologists. Nevertheless, future research endeavours should focus on broadening its scope to encompass various biopsy techniques, including cryobiopsy, to enhance the model's versatility and real‐world applicability. Additionally, incorporating data from diverse medical sources and facilities would provide a more comprehensive assessment of the model's performance and robustness.

In summary, we developed a multimodal AI algorithm encompassing both CT and histopathological images to accurately recognise UIP in patients with resected lung specimens. The extensive validation process, including comparisons with expert pathologists and general practitioners, highlights the significant potential of this AI tool in enhancing diagnostic capabilities and confidence among pathologists, even in cases where specialised expertise is limited. We believe that the model's performance in improving diagnostic accuracy and consistency signifies a critical step toward more standardised and reliable diagnoses in the field of ILD and that beyond the context of UIP diagnosis, this multimodal approach holds promise for addressing the complexities of other intricate medical conditions.

## Author Contributions

**Kris Lami:** conceptualization (equal), formal analysis (supporting), project administration (supporting), validation (supporting), visualization (equal), writing – original draft (lead), writing – review and editing (lead). **Mutsumi Ozasa:** conceptualization (equal), data curation (equal), formal analysis (equal), investigation (equal), project administration (supporting), validation (supporting), visualization (equal), writing – original draft (supporting), writing – review and editing (equal). **Xiangqian Che:** formal analysis (lead), software (lead), visualization (supporting), writing – original draft (supporting), writing – review and editing (equal). **Wataru Uegami:** conceptualization (supporting), data curation (supporting), formal analysis (supporting), project administration (supporting), software (supporting), visualization (supporting), writing – review and editing (supporting). **Yoshihiro Kato:** formal analysis (equal), software (equal), writing – review and editing (supporting). **Yoshiaki Zaizen:** data curation (equal), formal analysis (supporting), investigation (equal), writing – review and editing (supporting). **Naoko Tsuyama:** data curation (equal), formal analysis (supporting), investigation (equal), writing – review and editing (supporting). **Ichiro Mori:** data curation (equal), formal analysis (supporting), investigation (equal), writing – review and editing (supporting). **Shin Ichihara:** data curation (equal), formal analysis (supporting), investigation (equal), writing – review and editing (supporting). **Han‐Seung Yoon:** data curation (equal), formal analysis (supporting), investigation (equal), writing – review and editing (supporting). **Ryoko Egashira:** data curation (equal), formal analysis (supporting), investigation (equal), writing – review and editing (supporting). **Kensuke Kataoka:** data curation (equal), formal analysis (supporting), investigation (equal), writing – review and editing (supporting). **Takeshi Johkoh:** data curation (equal), formal analysis (supporting), investigation (equal), writing – review and editing (supporting). **Yasuhiro Kondo:** data curation (equal), formal analysis (supporting), investigation (equal), writing – review and editing (supporting). **Richard Attanoos:** data curation (equal), formal analysis (supporting), investigation (equal), writing – review and editing (supporting). **Alberto Cavazza:** data curation (equal), formal analysis (supporting), investigation (equal), writing – review and editing (supporting). **Alberto M. Marchevsky:** data curation (equal), formal analysis (supporting), investigation (equal), writing – review and editing (supporting). **Frank Schneider:** data curation (equal), formal analysis (supporting), investigation (equal), writing – review and editing (supporting). **Jaroslaw Wojciech Augustyniak:** data curation (equal), formal analysis (supporting), investigation (equal), writing – review and editing (supporting). **Amna Almutrafi:** data curation (equal), formal analysis (supporting), investigation (equal), writing – review and editing (supporting). **Alexandre Todorovic Fabro:** data curation (equal), formal analysis (supporting), investigation (equal), writing – review and editing (supporting). **Luka Brcic:** data curation (equal), formal analysis (supporting), investigation (equal), writing – review and editing (supporting). **Anja C. Roden:** data curation (equal), formal analysis (supporting), investigation (equal), writing – review and editing (supporting). **Maxwell Smith:** data curation (equal), formal analysis (supporting), investigation (equal), writing – review and editing (supporting). **Andre Moreira:** data curation (equal), formal analysis (supporting), investigation (equal), writing – review and editing (supporting). **Junya Fukuoka:** conceptualization (lead), data curation (equal), funding acquisition (lead), investigation (equal), methodology (equal), project administration (lead), supervision (lead), validation (lead), visualization (equal), writing – review and editing (equal).

## Ethics Statement

The study received approval from the Institutional Review Board of Nagasaki University Hospital (Approval number 22092203) to develop a multimodal AI system for accurately diagnosing UIP using a retrospective case collection approach. Informed patient consent was waived.

## Conflicts of Interest

Xiangqian Che and Yoshihiro Kato are employees of Strategic AI Group, Future Corporation. Other authors have no relevant conflicts to disclose.

## Supporting information


**Data S1.** Supporting Information.

## Data Availability

The data supporting this study's findings are available on reasonable request from the corresponding author (J.F.). The data are not publicly available due to hospital regulations.
