# Supplementary material for: Enhancing Interstitial Lung Disease Diagnoses Through Multimodal AI Integration of Histopathological and CT Image Data
Source: Respirology. 2025 Apr 2;30(8):726–35. doi: 10.1111/resp.70036 (PMC12321693; doi:10.1111/resp.70036)
Supplement: Supplementary file 1 — Data S1. Supporting Information. [file RESP-30-726-s001.docx]

**SUPPLEMENTAL MATERIALS**

**Enhancing Interstitial Lung Disease Diagnoses Through Multimodal AI Integration of Histopathological and CT Image Data**

**METHODS**

1. **Features extraction of CT-AI that recognizes UIP**

The radiological counterpart of the multimodal AI model is subsequently referred to as CT-AI. For its conception, CT images from 74 consecutive patients who had been diagnosed with chronic interstitial pneumonia and had undergone either VATS or transbronchial lung cryobiopsy (TBLC) were gathered. These CT images were consistently characterized by 0.5-0.6mm thin slices of the chest, captured using multiple scanner models (Aquilion Prime SP and Aquilion ONE, Canon Medical Systems, Tustin, CA; Toshiba Aquilion 16 and Toshiba Aquilion 64, BC Technical, West Jordan, UT; SOMATOM Definition Edge, Siemens Healthcare, Victoria, Australia). To facilitate the image processing, the lungmask library was used to perform the image segmentation exclusively on the lung field.^1^ Subsequently, the recognized lung field was divided into 32x32 pixels with a 48x48 pixels stride, acquiring a total of 574,977 patches (Figure 3-1).

Employing an automated, self-supervised learning methodology, we harnessed the SimSiam library for feature extraction from the CT images.^2^ This resulted in the transformation of the patches into feature vectors comprising 2,048 numerical attributes via the forward-propagation of the feature extractor (Figure 3).

Following this, a selection of 74,970 feature vectors patches were subjected to spherical k-means clustering, culminating in the subdivision of 120 clusters. These clusters were then subject to classification by two radiologists with specialization in ILD (Y.Z. and M.O.) into 8 distinct patterns (other cyst, non-UIP consolidation/ground glass opacity, non-UIP crazy paving, non-UIP GGO, non-UIP elastosis/other, early UIP, UIP reticular/consolidation/UIP reticulo-GGO, UIP HC) ((Figure 3-3). This classification was driven by an assessment of image features and their spatial localization within the lung field. The lung field was subdivided into 12 regions based on distinct anatomical divisions, including left-right, upper-middle-lower, and inner-outer sections, to facilitate feature extraction from CT images. To optimize the model for machine learning while avoiding an excessive number of features, a refined approach was employed. First, the proportions of eight distinct patterns were calculated across the upper, middle, and lower lung regions, yielding 24 features (3 regions x 8 patterns). Next, an additional feature was generated by calculating the difference in the proportion of normal lung between the left and right regions (n=1). Finally, the spatial variations in UIP patterns between the inner and outer regions were accounted for by adding three features representing the differences in these patterns across the upper, middle, and lower regions (n=3). This method resulted in a total of 28 features, providing a comprehensive representation of the lung’s anatomical and pathological characteristics (Figure 3-4).

1. **Creation of CT-AI that recognizes UIP**

The model weights are initialized by the SimSiam library, pretrained ResNet50 model. Due to the small size of the input patch, only the stride of the max pooling after the stem and the third block are 2, the stride of the rest parts is set to 1.

The other details of the model training are the same as SimSiam. The hyperparameters during training are presented in Supplemental Table 1.

Supplemental Table 1. Hyperparameters for the CT-AI training

| Epoch | 100 |
| --- | --- |
| Batch size | 128 |
| Optimizer | SGD |
| Momentum | 0.9 |
| Weight decay | 0.0001 |
| Learning rate | 0.005 (Cosine decay) |

Feature vectors patches used for the training underwent data augmentations as part of the SimSiam algorithm. Data augmentation techniques are summarized in Supplemental Table 2.

Supplemental Table 2. Data augmentation techniques

| Random min CT value (WL-WW/2) | [-1500, -1231] |
| --- | --- |
| Random max CT value (WL+WW/2) | [0, 401] |
| Random resize | [0.9, 1.1] |
| Random aspect ratio | [0.8, 1.25] |
| Random rotate | [-20°, 20°] |
| Random rotate by 0°, 90°, 180°, 270° | Probability = {0.25, 0.25, 0.25, 0.25} |
| Random color jitter | Probability = 0.8 |
| Random brightness | [0.6, 1.4] |
| Random contrast | [0.6, 1.4] |
| Random saturation | [0.6, 1.4] |
| Random hue | [0.9, 1.1] |
| Random Gaussian blur | Probability = 0.5 |
| Sigma | [0.1, 0.5] |
| Random horizontal flip | Probability = 0.5 |

The parameters of the Random Forest model of the CT-AI used for the prediction of the presence of UIP are as follows.

Supplemental Table 3. Random Forest model for the CT-AI parameters

| Number of trees | 25 |
| --- | --- |
| Maximum depth of the tree | 6 |
| Number of features to consider in split | 4 |

1. **Creation of the multimodal AI model combining histopathological and CT images**

For the creation of the multimodal AI model combining histopathological and CT images, the Random Forest model was also used, with the parameters given below:

Supplemental Table 4. Random Forest model for the multimodal AI parameters

| Number of trees | 40 |
| --- | --- |
| Maximum depth of the tree | 4 |
| Number of features to consider in split | 5 |
